# Understanding variability: the role of meta-analysis of variance

**DOI:** 10.1017/S0033291724001971

**Published:** 2024-09

**Authors:** Oliver D. Howes, George E. Chapman

**Affiliations:** 1 Department of Psychosis Studies, Institute of Psychiatry, Psychology & Neuroscience, King's College London, London, UK; 2 Faculty of Medicine, MRC Laboratory of Medical Sciences, Imperial College London, London, UK; 3 Division of Psychiatry, Faculty of Brain Sciences, University College London, London, UK

**Keywords:** clinical trials, coefficient of variation ratio, heterogeneity, meta-analysis, psychopharmacology, variability, variance, variance ratio

## Abstract

Meta-analyses traditionally compare the difference in means between groups for one or more outcomes of interest. However, they do not compare the spread of data (variability), which could mean that important effects and/or subgroups are missed. To address this, methods to compare variability meta-analytically have recently been developed, making it timely to review them and consider their strengths, weaknesses, and implementation. Using published data from trials in major depression, we demonstrate how the spread of data can impact both overall effect size and the frequency of extreme observations within studies, with potentially important implications for conclusions of meta-analyses, such as the clinical significance of findings. We then describe two methods for assessing group differences in variability meta-analytically: the variance ratio (VR) and coefficient of variation ratio (CVR). We consider the reporting and interpretation of these measures and how they differ from the assessment of heterogeneity between studies. We propose general benchmarks as a guideline for interpreting VR and CVR effects as small, medium, or large. Finally, we discuss some important limitations and practical considerations of VR and CVR and consider the value of integrating variability measures into meta-analyses.

Meta-analyses are generally considered to be the highest level of evidence and typically report mean differences between groups in outcomes of interest. Here we illustrate the importance of also understanding group differences in outcome variability and describe a method for approaching this meta-analytically.

Meta-analyses of randomized controlled trials (RCTs) of antidepressants in major depression find around a two-point greater reduction in symptom severity, as per the Hamilton Depression Rating Scale (HDRS), in the drug than placebo group (Hengartner, Jakobsen, Sørensen, & Plöderl, 2020; Jakobsen et al., 2017). Imagine two such trials – of drug A and drug B – both showing the same two-point greater improvement in HDRS with drug treatment than placebo. In the trial with drug A, the combined standard deviation of the treatment and placebo groups (i.e. pooled SD) is four points, which gives a medium treatment effect size (Cohen's *d* = 0.5) (Hengartner & Plöderl, 2018). Meanwhile, for drug B, the pooled SD is 10 points, which gives a small treatment effect size (*d* = 0.2) (Hengartner & Plöderl, 2018). Patients and clinicians might conclude that drug B offers a poor treatment effect on average and opt for drug A instead.

However, what if we were more interested in the chance of a drug greatly improving symptoms than its average treatment effect? Consider that we are interested in the proportion of patients who show a large (*d* *=* 1.0) antidepressant response – previously defined as an eight-point improvement in HDRS over placebo (Hengartner & Plöderl, 2018). In Fig. 1 below, we have plotted the distribution curves for drug A and drug B and have calculated (using EasyCalculation.com's Bell Curve Calculator; retrieved from: https://easycalculation.com/statistics/bell-curve-calculator.php.) the proportion of patients with at least an eight-point improvement. Whilst the mean benefit above placebo is two points for both drugs, the proportion of treated patients showing at least an eight-point greater improvement is around 7% for drug A and around 27% for drug B. The greater variability of drug B's treatment effect means that nearly three times more patients show a large effect size reduction in HDRS scores over placebo, with an odds ratio of almost five.

**Figure 1.**
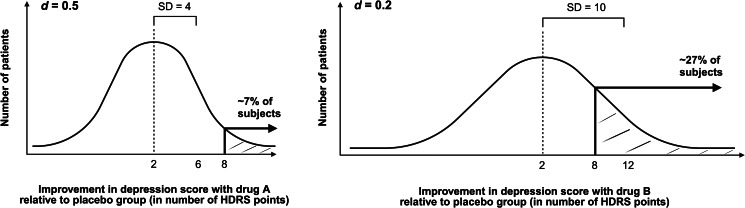
Sketches of hypothetical normal distributions of antidepressant responses to drug A and drug B, respectively. Hamilton Depression Rating Scale (HDRS) data (mean change and standard deviation) and effect sizes (Cohen's *d*) are taken from Hengartner & Plöderl, 2018. Figure created with BioRender.com.

Whilst our example compares two individual trials to illustrate the relationship between mean effect, outcome variability, and the frequency of extreme observations, similar effects of outcome variability may also be seen at the meta-analytical level. This is especially important to consider for meta-analyses that do not find group differences in mean outcomes. Subgroups of patients may demonstrate clinically meaningful responses to treatment not captured in comparisons of mean outcomes, but which would be reflected in measures of group variability. Whilst the presence of subgroups may be observed in raw trial data (e.g. if a probability distribution is multimodal), such data are seldom available in research articles, especially meta-analyses.

The ratio of the variance of an outcome measure in one group to that in another (variance ratio, VR) can be used to compare group variability meta-analytically (Hedges & Nowell, 1995). However, variance (the average of the squared differences of all data from the mean) is rarely reported in studies, whereas SD (the square root of the variance) is. Thus, the unbiased SD (SD adjusted for group differences in sample size) can be used to calculate the natural logarithm of VR, lnVR, as follows (McCutcheon et al., 2021; see also Nakagawa et al., 2015):
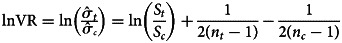
where, in the treatment *t* and control *c* groups, respectively: 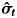 and 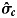 are unbiased estimates of the population SD, *S*_*t*_ and *S*_*c*_ are the sample SDs, and *n*_*t*_ and *n*_*c*_ are the sample sizes.

Variance is often directly proportional to a fractional index of the mean (that is, when the mean is raised to the power of a fraction, e.g. *x*^2/3^) (Taylor, 1961). Where this is the case, the SD scales with the mean, such that when mean*_t_* exceeds mean*_c_*, SD*_t_* is greater than SD*_c_*, in proportion to the difference in means. This phenomenon has now been observed in hundreds of biological systems and is perhaps driven by some common ‘context-independent’ mechanism (Giometto, Formentin, Rinaldo, Cohen, & Maritan, 2015). Thus, observed differences in group variability could be due to, or exaggerated by, mean scaling of variance. To avoid mean scaling effects, the natural logarithm of the coefficient of variation ratio (CVR), lnCVR, can be used (McCutcheon et al., 2021; see also Nakagawa et al., 2015), where:
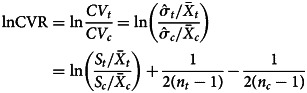


where 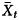 and 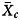 are sample means for the treatment and control groups, respectively. CVR circumvents scaling effects of the mean on SD by controlling for the mean differences between groups.

lnVR and lnCVR can be backtransformed to give VR or CVR, where a VR or CVR greater than one indicates increased variability in the treatment than control group, and vice versa. Modest variability ratios may be interpreted as percentage differences, e.g. a VR of 0.97 approximates a 3% group difference in variability (Winkelbeiner, Leucht, Kane, & Homan, 2019); however, this approximation may be less accurate when VR or CVR is large. We have described these formulae as applicable to reviews of RCTs but they are equally useful for meta-analyses of other study types (such as case–control studies of striatal dopamine function in schizophrenia; Brugger et al., 2020).

No guidelines yet exist for the interpretation of variance ratios. We suggest this could be addressed using an approach similar to Jacob Cohen's interpretation of standardized mean differences, *d* (Cohen, 1988). Cohen proposed that a *d* requiring measurement to detect, such as the mean standing height difference between groups of 15- and 16-year-old women (~1 cm, *d* *=* 0.2), is small; a *d* just about perceptible to the naked eye, such as the mean height difference between groups of 14- and 18-year-old women (~2.5 cm, *d* *=* 0.5), is medium; and a *d* that is easily perceptible, such as the mean height difference between groups of 13- and 18 year-old women (~4 cm, *d* = 0.8), is large.

Although these benchmarks are a helpful guide, Cohen was clear that grading a given effect as small, medium, or large should be based on the question at hand. For instance, in a small-scale RCT of a new antidepressant medication, a *d* less than 0.2 might represent an unsatisfactory treatment effect. Meanwhile, in epidemiological studies, similar effect sizes can have profound effects at the population level. For example, Carey, Ridler, Ford, and Stringaris (2023) suggest that a small (*d* = 0.14) increase in depressive symptom scores following the COVID pandemic may have resulted in as many as 160 000 excess cases of adolescent depression.

Using data analogous to Cohen (1988) from the Centres for Disease Prevention and Control National Health and Nutritional Examination Survey (NHANES) 2001–2002 (CDC, 2002), we find that the SD of standing heights of age groups of US women is ~0.5 cm greater in 13- than 18-year-old women, for which VR ≈ 1.19; ~2.5 cm greater in 8- than 13-year-old women, for which VR ≈ 2.01; and ~3.3 cm greater in 8- than 25-year-old women, for which VR ≈ 2.63. We therefore propose that a VR or CVR around 1.2 is considered small, around 2.0 is considered medium, and around 2.6 is considered large. Whilst this provides general benchmarks, we echo Cohen's caution in relation to *d*: that what is deemed to be a small, medium, or large effect should ultimately depend on the issue under consideration.

There are some important considerations for the interpretation of VR and CVR. We must first distinguish between variability and heterogeneity. VR and CVR are measures of the dispersion of study data relative to their mean. In contrast, heterogeneity describes the extent to which effect sizes differ across studies included in a meta-analysis (Higgins, Thompson, Deeks, & Altman, 2003). Heterogeneity is commonly assessed using the *Q* or *I*^2^ statistics, which indicate whether differences in effect sizes across studies are due to chance.

Second, a VR or CVR greater than one does not necessarily reflect greater variability in the patient/treatment group. It could, instead, reflect homogeneity in the comparator group, perhaps following recruitment of unrepresentatively healthy controls (i.e. volunteer bias) (Brugger et al., 2020). Similarly, exclusion of some patients could artificially reduce VR and CVR (McCutcheon et al., 2021). This may be an unintended effect of study exclusion criteria (e.g. excluding patients with treatment resistance, polypharmacy, or comorbidities) or recruitment strategies (e.g. excluding severely unwell individuals by solely recruiting outpatients).

Third, a VR or CVR equal to one may conceal mathematically cancelling group deviations, such as a bimodal distribution of non- and ultra-good responders (McCutcheon et al., 2021). It could also represent treatment-by-participant effects, which vary dependent on environmental, rater, and statistical factors (Winkelbeiner et al., 2019), coincidentally giving equal variances in both groups (Plöderl & Hengartner, 2019). It should also be appreciated that using total scores for a given scale might hide variability in specific symptom domains within the scale. This may be overcome by meta-analyzing the variability of individual items or subscale scores (McCutcheon et al., 2021). Furthermore, CVR is only valid for positive values of scales with a true zero point, so cannot be used for interval rating scales nor mean change values unless they are first converted to a ratio scale (Homan et al., 2021).

Finally, there may be more specific considerations related to the topic under study. Of note, in a meta-analysis of RCTs of antipsychotic medication in schizophrenia, McCutcheon et al. (2021) reported significantly lower variability of treatment response in patients receiving antipsychotics than in those receiving placebo. However, a re-analysis of the same 17 202 subjects found significantly higher variability of treatment response in patients receiving active treatment (McCutcheon et al., 2022). The authors reason that the former meta-analysis relied upon an invalid assumption that treatment and placebo effects are positively correlated. This was corrected in the later meta-analysis, in which the authors use patient- and study-level data to show that treatment and placebo effects are, in fact, negatively correlated (McCutcheon et al., 2022).

In summary, using variance ratios to synthesize group differences in variability provides important additional information to refine conclusions of meta-analyses of mean differences. This relatively novel meta-analytical approach has the potential to provide robust new insights into psychiatric and other illnesses, in terms of biology, treatment response, and other outcomes. Variability ratios of 1.2, 2.0, and 2.6 can be considered small, medium, and large, but interpretation must always consider the specific question at hand.

## Data Availability

For the purpose of open access, this paper has been published under a creative common licence (CC-BY) to any accepted author manuscript version arising from this submission.
